# Do Simulated Hospital Admissions Reflect Reality? A Qualitative Study of Volunteer Well-Being During a 24-Hr Simulated Hospitalization

**DOI:** 10.1177/19375867211020682

**Published:** 2021-06-09

**Authors:** Merlijn Smits, Yassin Eddahchouri, Pleun Meurs, Sharon M. Nijenhuis, Harry van Goor

**Affiliations:** 1 Department of Surgery, 6034 Radboud University Medical Center, Nijmegen, the Netherlands

**Keywords:** evidence-based design, patient room design, healthcare design, qualitative research, outcomes–patient, hospital, patient-person-centered care

## Abstract

**Objectives:**

This study aims to delineate if and how healthy volunteers admitted to simulated care can aid in understanding real well-being experiences of in-hospital surgical patients.

**Background:**

Scientific research is necessary to understand the mediating effect of healthcare design on patient outcomes. Studies with patients are, however, difficult to conduct as they require substantial funding, time, and research capacity, and recovering patients are often not willing or able to participate. If studies conducted with volunteers provide similar findings, such studies might serve as fruitful alternatives for future research.

**Method:**

A multimethod study was conducted between July 2017 and December 2017 with 17 volunteers who underwent a 24-hr simulated inpatient postsurgical care protocol. Data on value experiences, norms, and design requirements for an optimal healing environment were collected via diaries and semi-structured value-oriented interviews, focused on the values of spatial comfort, privacy, autonomy, sensory comfort, safety and security, and social comfort. Volunteers’ outcomes were compared to prior literature on similar patients’ outcomes.

**Results:**

Volunteers seem to experience their healing environment similarly to patients with regard to the values of spatial comfort, privacy, autonomy, sensory comfort, and social comfort related to contact with personnel and relatives. Less valuable insights were gained on the values of safety and security, and social comfort related to interaction with other patients, most probably due to the study design and because the participants did not truly experience a diseased bodily state.

**Conclusion:**

Simulated hospital admissions with volunteers provide a satisfactory alternative for studying real patient outcomes.

The mediating effects of the built environment have garnered increasing interest in the study of evidence-based design. Contemporary designers consider these mediating effects in their efforts to optimize user well-being. In healthcare design, physical spaces are called “healing environments” when they are designed to optimally contribute to the physical, mental, and social well-being of patients and healthcare personnel (Huisman et al., 2012).

The methodology of evidence-based healthcare design originates from the need to justify design choices related to the healing environment (Hamilton, 2003). This methodology is described by Ulrich et al. (2004, p. 24) as “a process for creating healthcare buildings, informed by the best available evidence, with the goal of improving outcomes.” To date, studies of environmental effects on patients’ experiences of well-being have most commonly used surveys. For example, several studies have asked patients to rate their stress resulting from elements of the environment (Karnik et al., 2014; Latha & Ravi Shankar, 2011; Pati et al., 2016). Other studies have considered patient satisfaction surveys regarding perceptions of the healing environment (MacAllister et al., 2016). More detailed explorations of patient experiences have been obtained from semi-structured interviews or context mapping exercises conducted with admitted patients. Our group used these methods to identify environmental factors related to postoperative recovery (Hesselink et al., 2020). Anåker et al. (2019) used these methods to investigate the experiences of stroke-unit patients. Other patient experience studies have directly involved patients in the design process by discussing design characteristics with patients to make evidence-based choices (Douglas & Douglas, 2004; Schreuder et al., 2016). In this vein, Lavender et al. (2020) conducted co-creation sessions with patients, and Patterson et al. (2017) showed simulated room prototypes to patients for feedback.

Despite the existence of many studies evaluating patients’ experiences of the healing environment, most insights can only be applied in specific settings. Given these studies’ potentially limited external validity, it has been argued that there are not enough studies to make general balanced evidence-based design decisions (Zborowsky & Bunker-Hellmich, 2010). In particular, original in-depth qualitative and quantitative studies of the effect of the healing environment on patients’ well-being are sparse, likely because it is challenging to properly and reliably conduct these studies. Among the challenges imposed by such studies are the requirements for substantial funding, time, and research capacity, which are barriers for healthcare institutions and healthcare designers. Further, measurement of outcomes and experiences is an involved and demanding task, particularly when imposed on patients who are still actively recovering from a disease or operation; this challenge frequently leads to refusals to participate or study dropouts (Agoritsas et al., 2011).

As an alternative to the use of patients, some scientists have recruited healthy volunteers for patient simulation studies. Andrade and Devlin (2015), for example, asked volunteers to imagine being a patient and questioned them their imagined needs. Similarly, Vincent et al. (2010) studied the effect of nature images on patients’ well-being by “admitting” volunteers to a simulated patient room. However, these studies bear only limited face and construct validity. Particular elements of the healing environment were studied for only a period of a few hours, mostly during daytime; to our knowledge, no study has subjected volunteers to simulated interactions with healthcare design elements and care processes of comparable duration and authenticity as those experienced by real patients. Indeed, the ability to involve healthy volunteers in authentic care processes to gain an understanding of patient experiences of well-being would facilitate the conduction of an entirely new branch of impactful healthcare design studies and would greatly expand our capacity to employ evidence-based decision making in healthcare design. This study aims to delineate if and how healthy volunteers can be used to better understand the inpatient experience of admitted surgical patients.

***Indeed, the ability to involve healthy volunteers in authentic care processes to gain an understanding of patient experiences of well-being would facilitate the conduction of an entirely new branch of impactful healthcare design studies and would greatly expand our capacity to employ evidence-based decision making in healthcare design.***

## Method

### Study Design

**Figure 1.**
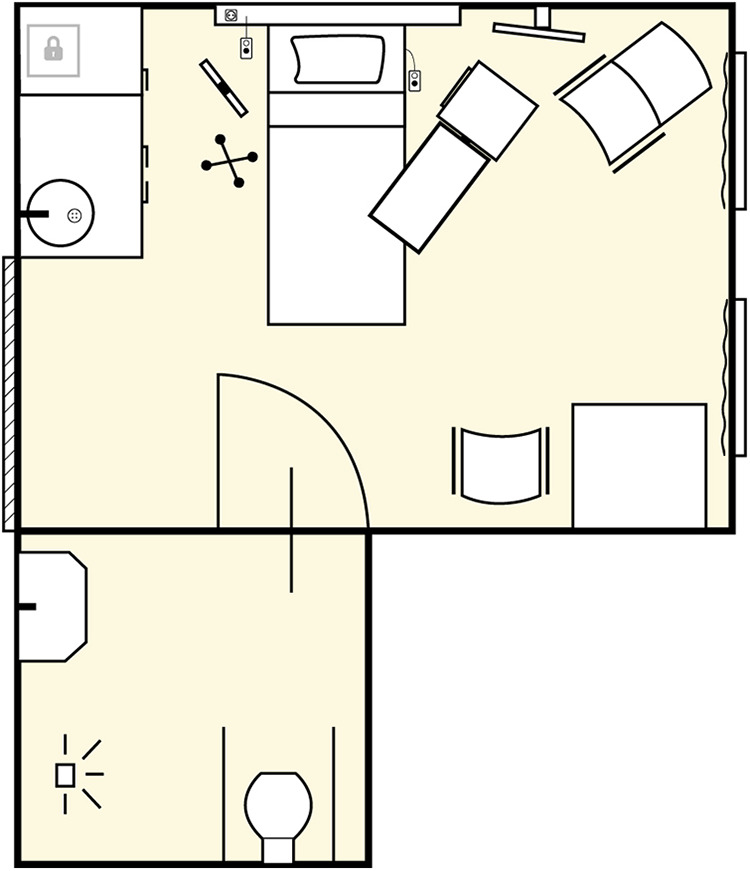
Map of the patient room for the 24-hr stay.

**Figure 2.**
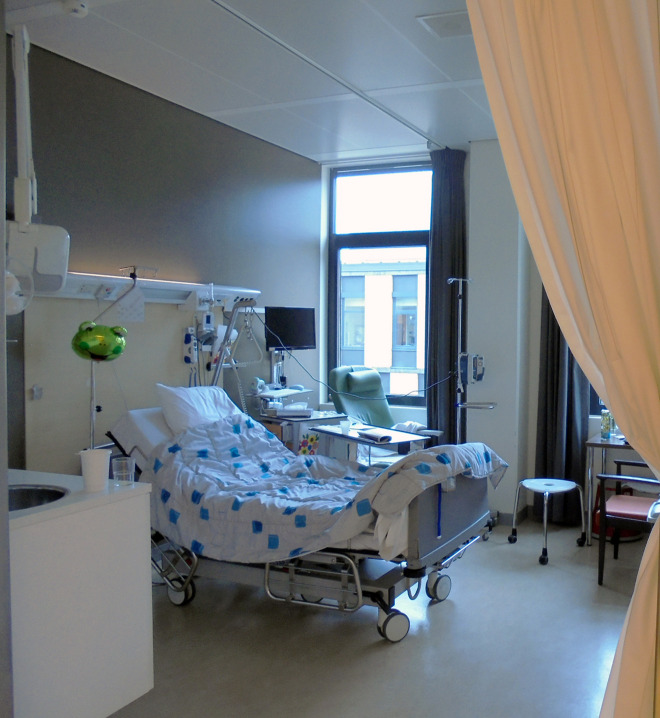
The patient room for the 24-hr stay.

A multimethod study design was adopted to understand if volunteer participants can aid in understanding real patients’ experiences and outcomes. Healthy volunteer participants were admitted as “patients” to a private room on the surgical ward of a Dutch university medical center for 24 hr. Figures 1 and 2 depict the patient room utilized for this study. The postoperative Day 2, nurse care protocol for a major abdominal cancer operation was followed for all admitted volunteers, mimicking conditions and interventions to which real patients would be subjected (see Online Appendix 1). Such interventions included an intravenous (IV) line attached to participants’ forearms and connected to a fluid bag and an IV pump, placebo medicines, three times daily vital sign measurements by nurses instructed to approach the volunteers as real patients, standard inpatient hospital food service, connection to a transcutaneous electrical nerve stimulation (TENS) device to simulate abdominal pain for 30 min several times per day, mobilization with a physiotherapist, and requirements to leave their patient rooms for a maximum of 30 min three times daily. Volunteers were prepared for admission by means of a booklet with information about the surgery ward (see Online Appendix 2). To study the experiences of the volunteers during admission, we utilized diaries and semi-structured value-oriented interviews conducted immediately after conclusion of the 24-hr stay.

The study was conducted over a 6-month period, during which time the care protocol remained unchanged. Standards for Reporting Qualitative Research (O’Brien et al., 2014) were followed to maximize validity. The local ethical commission approved the study (study ID: 2016-2899).

### Participants

Study participants were healthy adult volunteers, recruited by word-of-mouth advertising. Purposive sampling was used to ensure variability in participants’ gender, age, and highest received education to best reflect the actual patient population at the study ward. Study participants had to meet the following criteria: (1) age 18 years of age or older on the day the informed consent form was signed; (2) able to speak, read, and understand the Dutch language to familiarize themselves with the procedures of the study; and (3) agree to participate in the study program by giving oral and written informed consent.

### Data Collection

At the start of their hospitalization, participants were given a diary with open-ended questions probing their experiences, with a particular focus on sensory elements. Questions included “What did you do this afternoon?” “What do you hear and how do you feel about those sounds?” and “Draw your ideal patient room” (see Online Appendix 3). They were asked to reply to all questions during the admission. After 24 hr, participants returned the booklet and participated in a semi-structured value-oriented interview before discharge. A value-oriented interview is a well-validated method that has been repeatedly used to ascertain participants’ evaluative judgments and experienced values related to a certain design (Friedman & Hendry, 2019); in our study, this focus was applied to the healing environment.

The interview guide consisted of open-ended questions and probes derived from prior literature concerning six commonly found values known to be important for patients in their healing environments: spatial comfort, privacy, autonomy, sensory comfort, safety and security, and social comfort (College Bouw Zorginstellingen, 2008; Schreuder et al., 2016; see Online Appendix 4). Spatial comfort includes any physical aspect of the patient room that provides functional and personal support. Privacy includes factors that enable patients to have their “own” perceived space. Autonomy addresses the sensation of being in control and having the freedom to make one’s own decisions. Sensory comfort encompasses a set of pleasant sensory experiences resulting from the environment. Safety and security addresses patients’ experiences of feeling safe within the hospital environment. Finally, social comfort relates to pleasant interpersonal relationships and support.

Knowing already that these factors are of significant importance to real patients, we aimed to understand if volunteers experienced these values to a comparable degree as has been documented from real patients in the literature. The semi-structured interviews lasted 30–45 min and were conducted by a researcher with a medical degree (Y.E.).

### Data Analysis

Data obtained from the diaries and the semi-structured value-oriented interviews were systematically analyzed via thematic content analysis (Braun & Clarke, 2006). To minimize bias, two independent researchers with a background in industrial design engineering first analyzed all data separately (M.S., P.M.), followed by a thorough discussion and comparison of findings. Data derived from both diaries and interviews were brought together and categorized according to the six studied values. Each value was then translated into several norms, and subsequently into design requirements for an optimal healing environment, according to the methodology of Van de Poel (2013).

## Results

Seventeen volunteers with an average age of 44 (±13.2) were admitted in this study. Eight volunteers had been previously admitted to a hospital as a patient. Four participants were male, and 13 were female. Six participants—of whom four had been admitted in the past—positively replied to the question: “Did you feel like a patient over the past 24 hr?” The other 11 indicated that the lack of pain and stress related to surgery partially prevented them from having a genuine patient experience. In general, the volunteers provided a rich overview of how their optimal healing environment would function in terms of values, norms, and related design requirements. Design recommendations are illustrated below and listed in Table 1.

**Table 1.** Experienced Values, Norms, and Design Requirements by Healthy Volunteers Admitted 24 Hr to a Simulated Care Protocol.

Value	Norm	Design Requirement	Current Experience
Spatial comfort	Easy-to-use technology	The television should always be positioned in view of the patient.	The television is difficult to operate. It is heavy and placed out of reach.
		The television should have good quality sound and screen.	The quality of the sound and the screen of the television are low.
		The Wi-Fi connection should work seamlessly.	The Wi-Fi connection does not work properly.
		There should be a headphone with good quality sound (multi-bedroom) or a good sound system (private room).	The headphones have a low-quality sound.
		There is a stand for an iPad or laptop available.	It is difficult to use a laptop from out of bed.
		All control devices should be within reach from out of bed.	It is difficult to reach control devices (i.e., nurse-call button and bed hand control pendant) from out of bed.
		Patients can reach electric outlets from out of bed or wireless charging is available in reach.	It is difficult to charge a smartphone from out of bed.
		The patient room provides cableless devices, hides cables where possible, or combines cables where possible.	All cables of electronic devices make the room look cluttered.
	Comfortable furniture	The bedside table should be user-friendly: It should move around easily and provide sufficient space for personal items.	The bedside table is heavy, difficult to move around, provides too less space for personal items, and items easily fall off the table.
		The bed should be large enough. To prevent the sheets or patient from falling, it can be placed with one side to the wall.	The bed is too small. The sheets fall off the bed regularly.
		The chair should have a “warm” appearance.	The chair in the room has a “cold and clinical” appearance.
		There should be a comfortable chair for patients and visitors.	The chair in the room is not comfortable.
		There should be space in the bathroom to store personal items.	The bathroom does not provide space to store personal items.
	Comfortable interior design	The patient room should provide a warm and comfortable appearance.	The patient room feels “sterile, clinical, and cold.”
		There should be color in the patient room.	
		There should be nature in the patient room.	
	Positive distraction	The patient room should provide positive distraction by, for example, a PlayStation, a media device on the bed, or a digital screen on the wall allowing to show personal videos and photos.	It is boring in the patient room.
		The ward should provide activities to prevent boredom during the day.	
		There should be space for personal items in the patient room.	The patient room does not feel like home.
	Pleasant view	Patients should be able to see nature from out of bed.	Looking outside provides a boring and confronting view on other buildings and other patients’ rooms.
		Patients should not be able to look into the patient room of other patients from out of the window.	
		There should be a large window.	The window is too small.
		The bed should be able to be turned toward the window view.	It is difficult to look outside from out of bed.
Privacy	Visual privacy	Patients should be able to lock the toilet door.	It is nice to be able to lock the toilet door.
		Patients should have a private room.	It is nice to have a private room.
		Patients should know when someone is entering the patient room. Hospital personnel could knock, provide a sign, or ring a bell.	Hospital personnel sometimes enters the patient room without providing a sign.
		Patients should not have a view on other patient rooms from out of the window. For example, by windows that allow for looking out but that hinder others from looking in.	It is possible to look into the patient room of other patients via the window.
		It should be possible to move the home trainer into the patient room.	Exercising on the home trainer in the hallway does not improve feelings of privacy.
	Auditory privacy	Sound should be muted by the design of the building so that personal conversations cannot be heard by others.	It is possible to hear personal conversations in other patient rooms or in the hallway.
Autonomy	Autonomy in the ambient environment	Patients should have a device that allows to control light, door, curtains, and temperature from out of bed.	It is impossible to control the light from out of bed.
			It is impossible to open or close the door from out of bed.
			It is impossible to open or close the curtain from out of bed.
			It is impossible to control the temperature from out of bed.
		Patients should be able to control the sunscreen.	The sunscreens cannot be controlled. They close automatically.
		Patients should be well informed about the use of all devices for controlling the ambient environment.	It is not always clear how to control the ambient settings.
	Autonomy in planning	Patients should be involved in the planning of the day.	Patients are often unaware of the day planning and want to be involved in the planning.
		Patients should be informed about what they can expect during their hospitalization. For example: provide information on beeping infusion pumps and medical checkups at night.	Patients are often unaware of what to expect during the hospitalization.
		Patients should be well informed about the activities offered by the hospital.	Patients are often unaware of all activities offered by the hospital.
		Information provision should be personalized.	Each individual has a different information preference.
	Autonomy in help request	It should be possible to differentiate between different types of help requests.	The nurse-call button cannot differentiate between different types of help requests.
		The nurse-call button should provide feedback on send help calls.	The nurse-call button does not indicate whether help calls are send.
	Autonomy in mobility	The infusion pump should be mobile.	The infusion pump hinders simple actions as getting a glass of water or visiting the toilet.
Sensory comfort	Light	There should be enough light during the daytime.	It is difficult to read a book during the daytime. There is not enough light.
		The light should be adjustable.	The light cannot be turned into warm light.
		Patients should be able to turn off all lights at night.	At night, light from the hallway and the medical devices hinders a quality sleep.
	Sound	The patient room should mute sounds to only background noise.	Subtle sounds from the hallway are pleasant. Louder conversations are experienced as irritating.
		(Medical) devices should not generate sounds or good earbuds or headphones should be facilitated.	The sound of medical devices, sunscreens, and clock is experienced as irritating.
		The infusion pump should be silent.	The sound of the beeping infusion pump is experienced as stressful.
	Smell	A smell neutralizer should be installed or fresh air should be able to enter the patient room.	The smell in the hospital might be disliked.
	Temperature	Patients should be able to control the temperature.	Experience of temperature differs greatly per individual.
	Taste	The food should be of high quality and variety, and delivered multiple times a day.	The food is of high quality and variety, and delivered multiple times a day. This is appreciated.
		Patients should be able to choose the preferred moment of food delivery.	It is not possible to choose the preferred moment of food delivery.
		Patients can order more portions of food per time.	The portions of the food are experienced as too small.
Safety and security		Patients should be able to hear background noise from the hallway.	The sounds from the hallway provide a sense of safety.
		Patients should have a good working nurse-call button.	The nurse-call button provides a sense of safety.
Social comfort	Contact with hospital personnel	Hospital personnel should be friendly.	Hospital personnel is friendly.
		The roles of hospital personnel should be clear to patients. Personnel’s attire might help in differentiating between roles.	The roles of hospital personnel are sometimes unclear.
		Patients should know when someone is entering the patient room. Hospital personnel could knock, provide a sign, or ring a bell.	Hospital personnel sometimes enters the patient room without providing a sign.
		The nurse-call button should indicate to whom a help request is send.	Patients are unaware to whom their help requests are send.
		It is not always necessary to receive personal help. Some questions might be answered via a text or video message.	Not all help requests require personal contact with a nurse.
	Contact with patients	Not applicable	Not applicable
	Contact with relatives	Patients should be able to be in contact with their family or friends.	Contact with family or friends is very much appreciated.

### Spatial Comfort

All experiences related to spatial comfort were categorized into five domains: easy-to-use technology, comfortable furniture, comfortable interior design, positive distraction, and pleasant view.

#### Easy-to-use technology

In general, participants appreciated the ability to watch television and listen to music in the patient room as a form of distraction. Several remarks were made suggesting making these, and other technologies, more user-friendly. Six participants, for example, indicated that the weight and current positioning of the television (placed on an extendable arm behind the bed) made locating the device problematic, especially from a lying or sitting position. Two participants would have preferred a faster Wi-Fi connection. One participant desired quality sound in the headphones provided for shared rooms or a sound system in private rooms. Another participant wished to have a bed stand to enable use of his laptop on the bed.

Comments frequently referenced an inability to reach control devices while in the bed. Four participants could not always reach the nurse-call button or the hospital bed hand control pendant, as the cable limited its positioning. Five participants complained about being unable to charge their smartphones from the bed, as the electric outlets were located out of reach. Finally, all the electronic devices’ various cables made the room look cluttered. One participant suggested that wireless functionality and charging would make the patient room more relaxing. “The electricity plugs are located too far from the bed to charge your phone” (P15).

#### Comfortable furniture

In an ideal healing environment, the participants preferred more comfort offered by room furniture. Five participants desired alterations to the bedside table; the current one was heavy, difficult to move around, provided too little space for personal items, and allowed items to easily fall off. Most participants liked the bed; three desired a larger bed since sheets seemed to fall off regularly. One participant suggested turning the bed from its head side to its long side against the wall to feel more protected. Two participants desired a chair with a better appearance and more comfort. While most participants did not mention the bathroom, one suggested providing more space to store personal items.

#### Comfortable interior design

All participants agreed on the need for a “warmer” appearance of the patient room. Participants described their rooms as “sterile,” “clinical,” and “cold.” Five participants would have appreciated more color on the walls. Other recommendations referred to improving the ceiling and floor, placing plants and artworks, and hiding cables and personal items for better appearance. Participants did not mention size and shape of the patient room or distance to the bathroom as element of their spatial comfort.

#### Positive distraction

All participants desired to experience a positive distraction during admission, for example, via the television. Several suggestions were made for alternative forms of positive distraction. Two participants wanted to decorate the room with postcards and photos. One participant wanted to join activities in the ward. Most suggestions related to digital technologies in the patient room, including gaming consoles, media devices for video streaming, and the ability to listen to music. Finally, one participant suggested equipping each room with a digital screen on the wall with the ability to display personal videos, photos, and postcards. “I would like to have a view on a large screen. I would personalize this screen to my preferences; from one color to videos that I like” (P3).

#### Pleasant view

The view from the patient room showed participants only buildings and views into other patients’ rooms. Thirteen participants disliked the view, believed the windows to be too small, or said that it was difficult to look outside when lying on the bed. Ideally, five participants preferred seeing nature and enough daylight throughout the day.

### Privacy

The participants ranked privacy as the second most important aspect of a stay in the hospital, surpassed only by good communication and contact with hospital personnel. Based on their descriptions of privacy, we were able to make a further delineation between visual and auditory privacy.

#### Visual privacy

Almost all participants experienced visual privacy positively. Fourteen participants indicated an acceptable level of visual privacy thanks to the private room they stayed in, the ability to close the room with a door or curtain, and the ability to lock the toilet. Nevertheless, multiple suggestions for improvement were made. First, hospital personnel sometimes walked into the patient room without providing a notification beforehand. Two participants liked them to knock on the door and one suggested to have a doorbell ringing automatically when someone enters. Second, two participants disliked being able to see into others’ patient rooms via the window, as it made them feel that they too were being watched. One participant suggested a window that allows for looking out but hinders others from looking in. Third, certain activities, such as exercising on a home trainer, are currently done in the hallway; one volunteer suggested it would improve visual privacy if the home trainer could be brought into the patient room.

#### Auditory privacy

All participants indicated having satisfactory auditory privacy thanks to their private room. However, six participants reported being able to hear personal conversations coming from the hallway or other patient rooms when their door was opened. These participants felt guilty for hearing personal conversations, especially conversations about others’ medical conditions, and wondered if others could hear them also. Closing the door reduced sound but made one volunteer feel locked-up. “I can hear them talking about medical conditions. It makes me feel guilty. I don’t think I should be able to hear personal medical information of other patients” (P10).

### Autonomy

While all participants favored autonomy during hospitalization, only one participant indicated that he actually felt autonomous. All others saw room for improvement in four domains: ambient environment, planning, help requests, and mobility.

#### Autonomy in the ambient environment

To follow the care protocol of a postsurgical patient, participants were asked not to leave their beds without a nurse’s permission. Being unable to mobilize, all participants had difficulties with regulating the ambient environment themselves. They had to ask the nurse for help with such basic elements as opening and closing the curtain or door, and changing the lighting or the temperature in the room. Five participants suggested having a control device close to their bed allowing for regulation of the ambient environment.

#### Autonomy in planning

In general, participants prefer to be better informed about their daily planning. Seven participants wanted to receive information earlier or before admission. Not being able to know what to expect and when induced a degree of stress for the volunteers. “I never knew what to expect exactly. The nurses said: we’ll come back. But when?” (P6). Suggestions included the use of an agenda with scheduled activities during the day, managing expectations regarding environmental experiences such as beeping infusion pumps and medical checkups at night, and providing information on the facilities in the hospital. At the same time, information overload should be prevented; one participant preferred to be less informed.

#### Autonomy in help requests

The current nurse-call system does not allow for differentiation between different types of help requests. Improving this system would improve participants’ experience of autonomy. Three participants felt guilty for pushing the button for perceived “simple” calls such as getting a glass of water or changing clothes, as “real” patients might be in a greater need of a nurse’s assistance. Another participant remarked that the call button does not indicate whether the help request has been received and acknowledged, inducing uncertainty. One participant felt lonely when waiting 20 min for help before finally finding out that the help request had never been sent. “Although everyone is very friendly, I feel guilty for pushing the help button. […] Together with the nurse, I filled a bottle with water, so that I don’t have to ask for it every time” (P10).

#### Autonomy in mobility

The infusion line attached to the pump greatly decreased mobility—and thereby the autonomy—of all participants. Simple actions as getting some water or visiting the toilet became difficult to undertake. One participant found visiting the toilet troublesome, so she decided to lower her fluid intake against instructions during the admission. All participants agreed that a wearable infusion pump would greatly benefit autonomy.

### Sensory Comfort

Sensory comfort was mostly analyzed from the experiences of the participants noted in the diaries. We were able to differentiate between comfort in light, sound, smell, temperature, and taste.

#### Light

In general, participants saw most room for improvement in sensory comfort by changing the current lighting conditions. All but one participant suggested such changes. Two participants would have wanted to dim their lights. One suggested providing a bedside lamp for reading books. At night, light from the hallway and the medical devices hindered a sleep for three participants. One participant who visited the toilet at night complained about the brightness of the bathroom lights.

#### Sound

Participants reported a large variety of sounds in the hospital. Some were comforting; others were experienced to be stressful. Subtle sounds from the hallway, such as walking staff, quiet conversations, or a buzz, made 12 participants feel comfortable and even feel safe. Louder conversations, however, were experienced by six participants as irritating and for some induced feelings of guilt when these were private discussions. These sounds were mostly heard when the door of the room was opened, which was preferred by two participants as it provided them with fresh air. Other sounds, such as the clock or the flapping sunscreens, were disliked by two participants. Six participants experienced the alarm of the infusion pump as highly stressful. When it started beeping at night, one participant said that he felt extremely guilty of being afraid to wake up patients in other rooms. Several suggestions on sound improvements were made relating to silent medical alarms, silent wheels of infusion pumps, silent doors, silent clocks, earbuds at night, and good headphones to mask unpleasant sounds. “I hear people talking, walking around, conversations between nurses and patients. It makes me feel calm and content. It provides me with a comfortable feeling that I am not alone” (P4; participant desired to have the door opened all day).

#### Smell

Generally, smell was not an important item for the participants. Only three participants mentioned smell in any capacity. For these individuals, the smell in the patient room was noted to be “typical,” “clinical,” and “uncomfortable.” They suggested changing the bathroom smell into a soap-like smell and to allow the patient room to be filled with fresh air once in a while, as they were unable to open the windows.

#### Temperature

Half of the participants mentioned temperature as an important aspect of sensory comfort. Eight participants preferred a different room temperature, of which five wanted a decrease in temperature and three desired an increase. Experiences differed between day and night. Sensory comfort could be improved by controlling temperature from the bed.

#### Taste

Currently, our hospital’s food service delivers patients with food options six times per day. All participants appreciated the service, variety, and quality of the food. Only a few suggestions were made on increasing the size of meals and delivering it at a later time.

### Safety and Security

Participants were specifically asked about their feelings of safety and security in the patient room during the interviews. None of them indicated feelings of being unsafe or insecure. The alarm button for help requests provided a sense of comfort, knowing there would be help any time when needed. Hearing quiet conversations in the hallway and other rooms helped alleviate feelings of not being alone and thus feeling safe. Although a locker was provided, participants did not use it.

### Social Comfort

Social comfort was marked as the most important value related to well-being in the patient room. We subcategorized into three categories: contact with hospital personnel, relatives, and other patients.

#### Contact with hospital personnel

During their 24-hr stays, participants engaged multiple times with hospital personnel and were very positive about the received “care.” Only a few recommendations were given. First, one participant could not identify the role of each caregiver entering the room, which created confusion and stress regarding what to expect from the interaction. A better distinction through caregiver attire was suggested. Second, two participants would prefer that caregivers knock on the door before entering the room and properly introduce themselves. Third, even though the participants liked to stay in contact with personnel during the day, one commented that some nurse visits were redundant. He suggested using a smartphone application as an alternative means of asking questions and receiving answers, hypothesizing that this would reduce the workload of the nurse and lower the threshold for asking questions. “It would be nice to contact the nurse with a short text message: ‘Am I allowed to visit the toilet on my own?’” (P16).

#### Contact with relatives

In general, good contact with relatives was much appreciated. Twelve of the 17 participants were visited by a relative during their 24-hr stay. Those who did not receive a visit communicated with relatives via phone calls or text messages. No design recommendations were given.

#### Contact with patients

There was no contact between the participants and other patients in this study. As participants were only occasionally allowed to leave their room, they did not meet with other patients. In addition, one participant indicated avoiding contact with real patients as that made him feel embarrassed. “It felt embarrassing to contact patients as a healthy volunteer pretending to be a patient” (P3).

## Discussion

To understand the relationship between well-being and the healing environment, we studied the mediating effects of the healing environment on fundamental values. To this end, healthy volunteers were admitted for a 24-hr period of simulated care. Experiences related to six important patient values were studied and translated into norms and design requirements for an optimal healing environment. This process allowed us to understand how the healing environment affects important elements of well-being and allows designers to make and prioritize design decisions based on their effects on values of well-being. In addition, this study showed that the healing environment extends beyond the physical environment (e.g. walls, outside view, furniture) and is, indeed, inclusive of elements such as interaction with technology, expectations regarding care protocols, and feelings toward others. In turn, value experiences seemed to rely on an interplay between the healing environment and one’s physical and mental condition. These findings indicate that future designers should follow a values-based design approach in the design of healing environments in order to increase the well-being of admitted patients.

To facilitate future studies of the healing environment, we aimed to understand if healthy volunteers admitted to simulated care can provide “real” patient experiences. When comparing the results of this current study with existing literature, it appears that healthy participants do indeed provide valuable design insights that are comparable to real patients’ values of spatial comfort, privacy, autonomy, sensory comfort, and social comfort related to contact with personnel and relatives (Devlin & Arneill, 2003; Herweijer-van Gelder, 2016; Hesselink et al., 2020; Ulrich, 1991). For spatial comfort, autonomy, and sensory comfort, the participants suggested design improvements such as better electronic equipment, control over the ambient environment, information about day planning, improved mobility, and improved lighting conditions. Participants believed their privacy and social comfort requirements were already met well. They experienced good visual and auditory privacy—mostly because they were admitted to private rooms—and had generally positive interactions with hospital personnel and relatives. Less valuable insights were gained on the values of safety and security, and social comfort related to interaction with other patients.

***When comparing the results of this current study with existing literature, it appears that healthy participants do indeed provide valuable design insights that are comparable to real patients’ values of spatial comfort, privacy, autonomy, sensory comfort, and social comfort related to contact with personnel and relatives.***

Good spatial comfort is important for patients to feel comfortable and reduce experiences of stress and pain (Dijkstra et al., 2006). Our healthy volunteers mostly allude to similar norms and design requirements as found in the literature. Furniture and interior design, for example, have been found to directly affect feelings of comfort (Patterson et al., 2017). The need for positive distractions is part of Ulrich’s theory of supportive design (Ulrich, 1991) and improves comfort. A good outside view reduces stress, and provides decoration and distraction, especially when showing nature (Devlin & Arneill, 2003). The norm expressed by our volunteers on easy-to-use technology has not yet commonly been described in other studies. This might be a consequence of our open study design and our relatively young study population; young participants may be more likely to utilize and suggest the use of digital technology, although the use of digital technology is rapidly increasing in the elderly.

Experiencing visual and auditory privacy is intrinsically an important factor for well-being in the healing environment (Yildirim & Yalcin, 2016). Some norms and design requirements for optimal privacy referenced by our volunteers are also found in the literature; among these are a private room with curtain and door, hospital personnel knocking before entering, and avoidance of overhearing personal conversations from the hallway (Bäck & Wikblad, 1998; Hesselink et al., 2020; Huisman et al., 2012).

Patients’ autonomy is a prominent topic in healthcare design. Patients often lack autonomy during admission (College Bouw Zorginstellingen, 2008), generating stress, passivity, depression, and reduced immune function (Ulrich, 1992). To increase autonomy, our volunteers referred to norms also found in literature: more control over the ambient environment, need for better involvement in planning, more control over help requests, and a desire to be more mobile (Devlin & Arneill, 2003; Herweijer-van Gelder, 2016; Hesselink et al., 2020; Lipson-Smith et al., 2019).

Sensory comfort is important for patients’ well-being, especially with regard to light, noise, and fresh air and their consequent impacts on mood, stress, sleep, and recovery (Herweijer-van Gelder, 2016; Schreuder et al., 2016; Van den Berg, 2005). Our volunteers expressed a need for better lighting conditions in the patient room and indicated that they experienced stress resulting from private conversations and sounds of medical devices, especially at night. Some desired fresh air in the room. The differences in volunteers’ experiences between day and night highlight the added value of our study design lasting 24 hr rather than only a few hours.

The need for safety and security was not apparent in our volunteer group; all participants indicated that they felt safe. The absence of a need for safety likely mostly results from the volunteers being healthy; disease inherently reduces feelings of safety and security, and the possibility of adverse events from medicine intake or surgery reduces these feelings even more. Although healthy volunteers seem not to experience great needs for safety/security, a few suggested norms relating to the value are found in literature: availability of a locker, an alarm button, and the sound of conversing hospital personnel (Hesselink et al., 2020; Schreuder et al., 2016).

Finally, the need for social comfort is often stressed as an important element of well-being in the healing environment (McLaughlan, 2018; Sakallaris et al., 2015). Positive interactions with hospital personnel and relatives clearly mattered for our volunteers. No design improvements were suggested related to patient room design to address the perceived needs of relatives, such as additional chairs and beds, or flexible interior design (Herweijer-van Gelder, 2016), probably because of the short admission period and the lack of any study participant’s relatives’ presence during the night. Further, the need to connect with other patients was not expressed in our study. While real patients seem to rely on the assistance of roommates in shared rooms (Ehrlander et al., 2009), none of the volunteers in this study spoke to other patients or felt the need to do so. This is likely a result of the private room and an absence of disease among our healthy volunteers.

Summarizing, volunteers mostly seem to allude to the same norms and design requirements as found in literature, excepting values of safety and security, and social comfort. In the latter domain, our study insights can be used to make design decisions regarding values in single-patient rooms for postoperative recovery.

Although we only briefly touched upon the relative importance of one value over others, there seem to be large differences between volunteers and patients. In our study, volunteers indicated positive contact with hospital personnel and good privacy to be the two values of most importance, while the values of safety and security mattered least during the simulated admission. This greatly contrasts with results gathered by Schreuder et al. (2016), who studied in detail what values matter most to real patients. These patients rated the values of safety and security, spatial comfort, and autonomy to be of the most importance and considered privacy to be of least importance for their well-being. It is conceivable that being diseased might not only affect the experience of the healing environment in terms of safety and security, and relationships with other patients but may additionally change the relative importance attached to values (e.g., with privacy becoming less important and safety and security becoming more important). An interesting future investigation could apply the same study design of Schreuder et al. to volunteers, to more reliably compare relative value importance between patients and volunteers, and to provide deeper insights in the effects of disease on different values.

### Implications for Future Practice

Our study adds to the understanding of the nuances of studying volunteer patients in comparison with “real” hospitalized patients. Our findings may orient healing environment researchers and designers to consider the following six areas in their decision making:

1. *Healthy volunteers versus patients.* Amid constant changes in healthcare design and ever-evolving healthcare technologies, there is a growing need for studies on patient experiences to aid in design decisions. Simulated admissions with healthy volunteers could reduce study load on recovering patients and facilitate a greater body of research. Importantly, however, it must be noted that healthy volunteers cannot provide genuine experiences related to the actual state of being diseased. This, for example, affects their feelings of safety and security, their need for peer support, and the importance they attribute to privacy. Future studies should, therefore, first consider if volunteers could meaningfully aid in the study and, second, if volunteers’ absence of disease might negatively impact the study objectives.
***Future studies should, therefore, first consider if volunteers could meaningfully aid in the study and, second, if volunteers’ absence of disease might negatively impact the study objectives.***
2. *Type of volunteers.* In our study, we used purposive sampling to guarantee a variety of participants. Yet, our volunteers were all Dutch middle-aged individuals. They were relatively young compared to the average hospitalized patient, which might have affected outcomes, such as the need for digital technology in the room. Future studies with volunteers should consider to what extent the volunteering population is reflective of the targeted patient population.
3. *Type of simulated care.* In our study, volunteers were admitted to the simulated care protocol of postsurgical patients. For example, participants were attached to an IV line, received placebo medicines, and were periodically subjected to simulated pain. Although we aimed at optimal simulation of the care protocol, it nonetheless diverged from real care in several important areas. For example, pain was only periodically experienced by the volunteers, and no adverse effects from medicine could be reasonably experienced. This might have affected the need for positive distraction (constant pain could increase the need for positive distraction) and feeling safe (the specter of adverse effects could affect feelings of safety). Future studies in volunteers should identify and simulate major elements of patients’ care protocols and consider how the difference between simulated and real care might affect study objectives.
4. *Study focus.* The healing environment extends beyond mere physical objects. Our study showed patients’ well-being also greatly depends on, for example, expectations, relations, and interactions with technology. Accordingly, healing needs seems to vary depending on the current physical and mental state of the patient, and changes over time. In future studies of the healing environment, these characteristics should not be overlooked.
***The healing environment extends beyond mere physical objects. Our study showed patients’ well-being also greatly depends on, for example, expectations, relations, and interactions with technology.***
5. *Study period.* The 24-hr stay provided richer information on volunteers’ experiences, compared to a stay of only a few hours. For example, it allowed us to identify experiences of the healing environment at night (e.g., worse sleep due to lights and sounds of medical devices) which differed from those during the day. The length of future study periods should be targeted to achieve all study objectives while keeping the study load for participants as minimal as possible.
6. *Focus on values*. It is a challenging task to optimally design healing environments. As values are fundamental to well-being, a values-based design approach can facilitate design decision making. At first glance, it seems difficult to target values in design as it remains vague what they exactly stand for. Yet, as we have shown here, an in-depth study of the lived experiences of volunteers or patients allows for relation of values to concrete norms and design requirements. Important in following a values-based approach to healing environments is to identify what specific values should be studied, and to identify to what relative extent the value in question adds to overall well-being, given that the importance of these values vary in the context of recovering patients.
***As values are fundamental to well-being, a values-based design approach can facilitate design decision making.***

## Conclusion

In this study, we admitted healthy participants to a simulated inpatient postsurgical care protocol. The volunteers provided reliable design requirement suggestions similar to real patients for the values of spatial comfort, privacy, autonomy, sensory comfort, and social comfort related to contact with personnel and relatives. Less valuable insights were gained on the values of safety and security, and social comfort related to interaction with other patients. In addition, the importance attributed to the values of privacy and safety and security seemed to differ between healthy volunteers and patients. The focus on values in this study allowed identification of the aspects of healing environments that improve the most fundamental elements of well-being, such as a need for user-friendly technology, positive distractions, and desire to control the environment from bed. This study has shown that volunteers can aid in understanding real patient experiences for the purposes of study design. Furthermore, a values-based focus generates optimal insights for design decision making. For future studies on healing environments, we provide several items for consideration to facilitate the generation of value for future patients.

## Implications for Practice

- The ability to involve healthy volunteers in authentic care processes to gain an understanding of patient experiences of well-being would facilitate the conduction of an entirely new branch of impactful healthcare design studies and would greatly expand our capacity to employ evidence-based decision making in healthcare design.
- Healthy volunteers admitted to a 24-hr simulated care protocol provide valuable design insights that are comparable to real patients’ values of spatial comfort, privacy, autonomy, sensory comfort, and social comfort related to contact with personnel and relatives.
- Healthy volunteers admitted to a 24-hr simulated care protocol do not experience a need for safety and security or a need to contact other patients, probably because the participants have not undergone surgery and do not feel sick.
- Future studies should first consider if volunteers could meaningfully aid in the study and, second, if volunteers’ absence of disease might negatively impact the study objectives.
- As values are fundamental to well-being, a values-based design approach can facilitate design decision making.

## Supplemental Material

Supplemental Material, sj-docx-1-her-10.1177_19375867211020682 - Do Simulated Hospital Admissions Reflect Reality? A Qualitative Study of Volunteer Well-Being During a 24-Hr Simulated Hospitalization Click here for additional data file. Supplemental Material, sj-docx-1-her-10.1177_19375867211020682 for Do Simulated Hospital Admissions Reflect Reality? A Qualitative Study of Volunteer Well-Being During a 24-Hr Simulated Hospitalization by Merlijn Smits, Yassin Eddahchouri, Pleun Meurs, Sharon M. Nijenhuis and Harry van Goor in HERD: Health Environments Research & Design Journal

Supplemental Material, sj-docx-2-her-10.1177_19375867211020682 - Do Simulated Hospital Admissions Reflect Reality? A Qualitative Study of Volunteer Well-Being During a 24-Hr Simulated Hospitalization Click here for additional data file. Supplemental Material, sj-docx-2-her-10.1177_19375867211020682 for Do Simulated Hospital Admissions Reflect Reality? A Qualitative Study of Volunteer Well-Being During a 24-Hr Simulated Hospitalization by Merlijn Smits, Yassin Eddahchouri, Pleun Meurs, Sharon M. Nijenhuis and Harry van Goor in HERD: Health Environments Research & Design Journal

Supplemental Material, sj-docx-3-her-10.1177_19375867211020682 - Do Simulated Hospital Admissions Reflect Reality? A Qualitative Study of Volunteer Well-Being During a 24-Hr Simulated Hospitalization Click here for additional data file. Supplemental Material, sj-docx-3-her-10.1177_19375867211020682 for Do Simulated Hospital Admissions Reflect Reality? A Qualitative Study of Volunteer Well-Being During a 24-Hr Simulated Hospitalization by Merlijn Smits, Yassin Eddahchouri, Pleun Meurs, Sharon M. Nijenhuis and Harry van Goor in HERD: Health Environments Research & Design Journal

Supplemental Material, sj-docx-4-her-10.1177_19375867211020682 - Do Simulated Hospital Admissions Reflect Reality? A Qualitative Study of Volunteer Well-Being During a 24-Hr Simulated Hospitalization Click here for additional data file. Supplemental Material, sj-docx-4-her-10.1177_19375867211020682 for Do Simulated Hospital Admissions Reflect Reality? A Qualitative Study of Volunteer Well-Being During a 24-Hr Simulated Hospitalization by Merlijn Smits, Yassin Eddahchouri, Pleun Meurs, Sharon M. Nijenhuis and Harry van Goor in HERD: Health Environments Research & Design Journal

Supplemental Material, sj-pdf-1-her-10.1177_19375867211020682 - Do Simulated Hospital Admissions Reflect Reality? A Qualitative Study of Volunteer Well-Being During a 24-Hr Simulated Hospitalization Click here for additional data file. Supplemental Material, sj-pdf-1-her-10.1177_19375867211020682 for Do Simulated Hospital Admissions Reflect Reality? A Qualitative Study of Volunteer Well-Being During a 24-Hr Simulated Hospitalization by Merlijn Smits, Yassin Eddahchouri, Pleun Meurs, Sharon M. Nijenhuis and Harry van Goor in HERD: Health Environments Research & Design Journal
