# Supplementary material for: Do Simulated Hospital Admissions Reflect Reality? A Qualitative Study of Volunteer Well-Being During a 24-Hr Simulated Hospitalization
Source: HERD. 2021 Jun 9;14(4):130–46. doi: 10.1177/19375867211020682 (PMC8597193; doi:10.1177/19375867211020682)
Supplement: Supplemental Material, sj-docx-1-her-10.1177_19375867211020682 - Do Simulated Hospital Admissions Reflect Reality? A Qualitative Study of Volunteer Well-Being During a 24-Hr Simulated Hospitalization [file sj-docx-1-her-10.1177_19375867211020682.docx]

**Appendix 1: Oncological surgery nurse care protocol healthy volunteers**

*Times are indications. Register in last column the actual time.*

Part 1 (10:30 – 12:00) Researcher

Part 2 (12:00 – 21:00) Nurse

Part 2

| **24-hours stay in private patient room (Room 66)** | | | **Date:** | ___-___-____ | |
| --- | --- | --- | --- | --- | --- |
| 10:25 | Contact with nurse / food service (food for care) to include healthy volunteer in daily meals. | | | | □ ____:____ |
| 10:30 | Reception of healthy volunteer at the surgery ward:   - Repeat study protocol - Inform about rules in hospital - Inform about diary and semi-structured interview | | | | □ ____:____ |
| 10:40 | **CRF6** STAI and **CRF7** VAS | | | | □ ____:____ |
| 10:45 – 11:15 | **CRF8** QST | | | | □ ____:____ |
| 11:15 | Connecting identification badge | | | | □ ____:____ |
| 11:15 | Check  vital signs | Nurse/ researcher: Define MEWS (see **MEWS protocol**), oxygen saturation, heartbeat, blood pressure (systolic), consciousness, temperature  Register data on **CRF11 of MEWS protocol.** | | | □ ____:____ |
| 11:30 –11:50 | Connecting IV line to forearm and connect to a fluid bag and an IV pump | Researcher: Connect following (with tape) to volunteer:   - Compression stockings - IV line and IV pump: **set up IV pump alarm at 13:30** - Bladder catheter: lower abdomen - Abdominal drain: upper abdomen - Epidural catheter: back | | | □ ____:____ |
| 12:00 –14:00 | Lunch | Food service | | |  |
| 12:00 | Medicine round | Nurse: provide volunteer with water and peppermint. | | | □ ____:____ |
| 13:30 | Sound - alarm | Alarm IV pump (5 minutes)  Nurse: turn off IV pump after 5 minutes. | | | □ ____:____ |
| 14:00-14:15 | Physiotherapy | Physiotherapist: breathing and mobility exercises. | | | □ ____:____ |
| 14:15 | Contact with nurse / food service (food for care) to include healthy volunteer in daily meals. | | | | □ ____:____ |
| 14:45 – 16:45 | Snack | Food service | | |  |
| 15:00 | Medicine round | Nurse: provide volunteer with water and peppermint. | | | □ ____:____ |
| 15:05 | Wound care | Nurse: replace band aid of all lines and drains.  **Set up IV pump alarm at 16:15.** | | | □ ____:____ |
| 15:15 – 15:45 | Mobilize | Nurse: encourage volunteer to walk ±30 minutes at ward. | | | □ ____:____ |
| **Own initiative** | Mobilize + Simulation pain | Researcher/nurse: 3/daily TENS during mobilization. Only at own request of volunteer. Volunteer calls researcher! | | | □ ____:____ |
| 16:15 | Sound - alarm | Alarm IV pump (5 minutes)  Nurse: turn off IV pump after 5 minutes. | | | □ ____:____ |
| 16:20 | Contact with nurse / food service (food for care) to include healthy volunteer in daily meals. | | | | □ ____:____ |
| 17:00 | Medicine round | Nurse: provide volunteer with water and peppermint. | | | □ ____:____ |
| 17:15 – 19:15 | Dinner | Food service | | |  |
| 17:55 | Check Healthpatch | Researcher/nurse: check if all data on the Healthpatch is saved. | | | □ ____:____ |
| 18:00 | Reminder diary | Researcher/nurse: remind volunteer to fill in diary for the afternoon.  Check if urinal needs to be emptied. | | | □ ____:____ |
| 18:55 | Contact with nurse / food service (food for care) to include healthy volunteer in daily meals. | | | | □ ____:____ |
| 19:00 | **CRF6** STAI and **CRF7** VAS | Researcher/nurse: ask volunteer to fill in CRF 6 and 7. | | | □ ____:____ |
| 19:00 – 20:00 | Snack | Food service | | |  |
| 20:00 | Physical care | Volunteer | | |  |
| 20:00  Part 3 (21:00 – 6:30) Nurse | Wound care | Nurse: replace band aid of all lines and drains. | | | □ ____:____ |
| 21:00 | Check  vital signs | Nurse: Define MEWS (see **MEWS protocol**), oxygen saturation, heartbeat, blood pressure (systolic), consciousness, temperature  Register data on **CRF11 of MEWS protocol.** | | | □ ____:____ |
| 21:15 – 21:45 | Mobilize | Nurse: encourage volunteer to walk ±30 minutes at ward. | | | □ ____:____ |
| **Own initiative** | Mobilize + Simulation pain | Researcher/nurse: 3/daily TENS during mobilization. Only at own request of volunteer. Volunteer calls researcher! | | | □ ____:____ |
| 22:00 | Medicine round | Nurse: provide volunteer with water and peppermint. | | | □ ____:____ |
| ±22:00 | Drinks | Nurse | | |  |
| 22:00 | Check Healthpatch | Researcher/nurse: check if all data on the Healthpatch is saved. | | | □ ____:____ |
| 22:05 | Reminder diary | Researcher/nurse: remind volunteer to fill in diary for the evening.  Check if urinal needs to be emptied. | | | □ ____:____ |
| 22:00 – 23:00 | Nighttime | Researcher: close door, turn off lights. | | | □ ____:____ |
| 00:30  Part 3 | Sound - noise | Researcher walks into patient room and wakes up volunteer. Try not to scare the volunteer! | | | □ ____:____ |
| 00:35 | Simulation pain | Volunteer: Connect TENS to abdomen for 15 minutes. | | | □ ____:____ |
| 2:30 | Check | Nurse: check if volunteer is sleeping (breathing?), volunteer may wake up ‘by accident’. **Set up IV pump alarm at 3:30.** | | | □ ____:____ |
| 3:30 | Sound - alarm | Alarm IV pump (5 minutes)  Nurse: turn off IV pump after 5 minutes. | | | □ ____:____ |
| 5:00 | Sound - noise | Sounds/talking on hallway 🡪 **discuss with nurses**. | | | □ ____:____ |
| 6:45  Part 4 (6:30 – 10.30) Nurse | Medicine round | Nurse: wake up volunteer and provide volunteer with water and peppermint. | | | □ ____:____ |
| 7:00 | Check  vital signs | Nurse: Define MEWS (see **MEWS protocol**), oxygen saturation, heartbeat, blood pressure (systolic), consciousness, temperature  Register data on **CRF11 of MEWS protocol.** | | | □ ____:____ |
| 7:10 | **CRF 9** LSEQ | Researcher: after MEWS **CRF9** LSEQ ask volunteer to reply to questions. | | | □ ____:____ |
| 7:10 | Reminder diary | Researcher/nurse: remind volunteer to fill in diary for the night.  Check if urinal needs to be emptied. | | | □ ____:____ |
| 7:15 | Contact with nurse / food service (food for care) to include healthy volunteer in daily meals. | | | | □ ____:____ |
| 7:15 | Check Healthpatch | Researcher/nurse: check if all data on the Healthpatch is saved. | | | □ ____:____ |
| 7:30 – 9:00 | Breakfast | Food service | | |  |
| 8:00 | Physical care | Volunteer | | |  |
| 9:00 | Wound care | Nurse: replace band aid of all lines and drains. | | | □ ____:____ |
| 9:15 | Visit MD | MD, nurse: discuss shortly the 24-hours. | | | □ ____:____ |
| 9:30 – 9:45 | Physiotherapy | Physiotherapist: breathing and mobility exercises. | | | □ ____:____ |
| 9:45 – 10:15 | Mobilize | Nurse: after physiotherapy, encourage volunteer to walk ±15-30 minutes at ward. | | | □ ____:____ |
| **Own initiative** | Mobilize + Simulation pain | Researcher/nurse: 3/daily TENS during mobilization. Only at own request of volunteer. Volunteer calls researcher! | | | □ ____:____ |
| 9:45 – 11:45 | Shake | Food service | | |  |
| 10:30 | Reminder diary | Researcher: remind volunteer to fill in diary for the morning.  Check if urinal needs to be emptied. | | | □ ____:____ |
| 10:30 | End of study | Researcher: Disconnect IV lines. | | | □ ____:____ |
| 10:30 – 10:35 | **CRF6** STAI and **CRF7** VAS | | | | □ ____:____ |
| 10:35 – 11:10 | **CRF8** QST | | | | □ ____:____ |
| 11:10 – 11:30 | **CRF10** Semi-structured value-oriented interview with volunteer. | | | | □ ____:____ |
| 11:30 | Disconnect Healthpatch🡪 **CRF 5** | | | | □ ____:____ |
| 11:30 | Thank volunteer and give present (gift card). | | | | □ ____:____ |

Part 5 (10:30 – 11.30) Researcher
