# Supplementary material for: Do Simulated Hospital Admissions Reflect Reality? A Qualitative Study of Volunteer Well-Being During a 24-Hr Simulated Hospitalization
Source: HERD. 2021 Jun 9;14(4):130–46. doi: 10.1177/19375867211020682 (PMC8597193; doi:10.1177/19375867211020682)
Supplement: Supplemental Material, sj-docx-2-her-10.1177_19375867211020682 - Do Simulated Hospital Admissions Reflect Reality? A Qualitative Study of Volunteer Well-Being During a 24-Hr Simulated Hospitalization [file sj-docx-2-her-10.1177_19375867211020682.docx]

**Appendix 2: surgery ward – home rules**

**About this ward**

The surgery ward is treating a large variety of patients. Patients can be treated for complex or simple diseases. Surgical procedures can be acute or planned. Other activities of importance for the surgery ward are scientific research and education of medicine students and surgeons.

12.500 new patients visit this surgery ward annually. Of them, 3.000 patients are admitted. 5.600 surgical procedures are executed. Patients vary in age from children to elderly. The ward is categorized in four subwards: Abdominal and Oncological surgery (abdominal and cancer surgery), Paediatric surgery, Trauma surgery (accident surgery), and Vascular and Transplant Surgery.

**Stay- general information**

We expect you to cooperate and behave. We expect respect and understanding for all patients and caregivers. Aggression, violence and threats are not accepted.

Three types of patient rooms can be found at the surgery ward: single patient rooms and shared patient rooms for two or four patients. Mostly, gender is not considered in admitting patients to rooms.

Patients are responsible for their own belongings. A small storage space is available for storing personal items.

We ask you to stay at the ward to be available for ‘medical’ procedures or other research activities.

Recovery is adjusted to personal needs and preferences of patients. Nevertheless, there is a general time schedule for each patient:

| Time | Activity |
| --- | --- |
| 06.30 - 07.30 AM | Medicine round  Checking vital signs: temperature, heartbeat, blood pressure, pain scores |
| 07.00 - 07.30 AM | Preparing patients for planned surgery at 8.00 AM |
| 07.30 - 07.45 AM | Nurse shift |
| 07.30 - 08.30 AM | Breakfast |
| 08.00 - 11.00 AM | Visit medical doctor |
|  | Assistance in physical care, wound care and mobilization |
|  | The food service will offer a variety of drinks |
| 11.45 - 1.00 PM | Eating breakfast  Medicine round  Checking vital signs: temperature, heartbeat, blood pressure, pain scores |
| 2.00 - 2.30 PM | Nurse meeting |
|  | The food service will offer a variety of drinks |
| 3.00 - 4.00 PM | Assistance in physical care, wound care and mobilization |
| 3.30 - 3.45 PM | Nurse shift |
| 5.00 - 6.00 PM | Eating dinner  Medicine round  The food service will offer a variety of drinks |
| 7.15 PM | Assistance in physical care, wound care and mobilization |
|  | Checking vital signs: temperature, heartbeat, blood pressure, pain scores |
| 8.00 - 10.00 PM | Medicine round |
|  | The food service will offer a variety of drinks for the night |
| 10.00 - 11.00 PM | Rest |
| 11.15 - 00.30 AM | Nurse shift |

**Mobility**

Mobility benefits recovery of patients. Our ward stimulates active recovery. Caregivers will stimulate patients to mobilize when possible during admission. Patients can, for example, mobilize by walking in their room or over the ward. It is also possible to do physical exercises. Sitting in a relaxation chair is preferred over sitting in the bed.

During your stay, we ask you to mobilize 30 minutes three times a day. You can leave bed and can make a walk over the ward. When you aim to leave bed, you must call the researcher. The researcher will now provide assistance to help you out of bed. You must wait before the researcher arrives before you leave bed. Apart from these three moments of mobilization, we ask you to stay in bed as a real patient would do, to optimize your experience as a patient. You do not have to ask for permission to visit the toilet.

**TENS during mobility**

When patients mobilize, they experience pain or feel uncomfortable due to the wounds of the surgery. To increase your feelings of being a patient, we would like to simulate this feeling of pain by means of TENS. TENS is a device that generates electrical pulses to stimulate the nerves. This generates feelings of discomfort. TENS is a scientifically proven safe method normally used to treat people with chronic pain. To use the TENS device, four stickers will be attached to your belly to which the TENS device is connected. The researcher will help you to install the TENS device. You are in control of the device; you can regulate what level of discomfort is appropriate.

**Diary**

We would like to obtain insight in your activities during your stay. We would like to ask you to report in a diary what activities you have done per part of the day (morning, afternoon, evening, night). You could also report what you see, feel or experience. Remarks for improvement of care might be added as well to the diary.

**Reporting fluid intake**

We would like to obtain insight in your fluid intake during the day. Could you report in the diary what you drink and how much you drink? Yoghurt, porridge and applesauce are also seen as fluids.

Contents:

1 cup 100 ml

1 glass with ear 150 ml

1 large cup 200 ml

1 dish with applesauce 100 ml

1 dish with yoghurt 150 ml

**Toilet visit**

Besides fluid intake, nurses also collect how much patients urinate. Ladies pie on a pot and men pie in a urinal. Both are in the bathroom. We ask you to use the pot or urinal to pie. The researcher will not measure how much ml is in the pot/urinal. The researcher or the nurse will empty the pot/urinal.

**Food service**

Our hospital provides food via the services offered by FoodforCare. Nutritional assistants will offer you food six times a day: shakes, smoothies, lunch, soup, warm food, salads, desserts, snacks, and sweet or savoury candy. You will be offered breakfast, lunch and dinner in appropriate portions. You are also offered shakes and snacks three times in between the meals. When you are still hungry, you can always ask for more food. The food service offers three different meals per round. Together with the nutritional assistant, patients normally discuss what meal meets best their preferences and needs. Taste, smell, colour, important nutritional ingredients (for example, proteins and energy) and portions are customized to the needs of patients.

Food is served at the following times:

09.45 - 11.45 AM Shake

12.00 – 2.00 PM Lunch including bread

2.45 - 4.45 PM Snack

5.15 - 7.15 PM Dinner

6.00 – 8.00 PM Dessert

7.00 - 8.00 PM Candy

Visitors can consume free coffee and tea provided at the ward.

**Visitors**

The ward does not have time slots for visitors. Visitors are welcome during the entire day. Yet, we advise you not to receive visitors before 10.00 AM or after 8.00 PM to improve rest of the other patients at the ward. We also advise a maximum of two visitors simultaneously. To ensure that each patient can receive visitors, we ask you to consider:

1. Visitors should not disturb the research activities. Discuss with the researcher when you can receive visitors.
2. To improve privacy, we can ask visitors to leave the room during care or research activities.
3. You can leave the patient room with your visitors. We would like to ask you to stay at the surgery ward.

**Personal belongings**

Unfortunately, personal belongings are sometimes stolen in the hospital. Please, take care of your own belongings. You are responsible for your own belongings. We advise you not to take valuable belongings, including jewellery, money and your driver licence, to the hospital. Do you still want to take valuable belongings to the hospital? Then hide them in a locked closet.

**Television and radio**

During your stay, you can watch television and listen to radio. There is a television close to your bed. Sound of radio and television is played via a headphone in shared rooms. In single bedrooms, patients are also allowed to play the sound out loud. Use of television and radio is free of charge.

**Internet**

You can use the internet of our hospital via your own devices. It is also possible to use internet via the screen besides your bed. Connect to the Wi-Fi network “Guests”.

**Photos, videos and sound recordings**

When you want to take photos, videos or sound recordings, always ask the persons that are recorded for permission. Keep in mind their privacy. Tell them what you aim to do with the photos, videos or sound recordings. In case of refusal, do not take photos, videos or sound recordings. It is also not allowed to record children.

The hospital makes use of security cameras to improve safety in the hospital. Signs show where the cameras are located. Recordings are saved shortly. Only in case of accidents, recordings are saved for the period of the investigation.

**Social media**

You are, of course, allowed to share via social media your opinion about our hospital. Yet, you are not allowed to share photos, videos and sound recordings of the hospital without permission of the people in the recordings. Always ask people for permission. Also respect the question of people to remove social media posts when the posts contain identifiable information. Be aware of what social media can evoke. Do not share anything that can hurt you, other patients or employees of the hospital.

**HealthPatch sensor**

During your stay, you will wear a sensor, the HealthPatch. This sensor is attached to your chest. It will constantly measure your vital signs, including your breathing, skin temperature and your mobility. You will not notice that the sensor is measuring. You can shower with the sensor. Yet, try not to use soap on the sensor. Also dry it carefully.

The sensor is connected wirelessly via an iPod. This iPod is located near your bed. You do not have to take the iPod when you leave your bed. Also, do not take the iPod to the bathroom. The iPod is not water resistant.

When the sensor does not anymore stick to the chest, try to attach it back. When the sensor falls of, do not throw it away and keep it for the researcher.


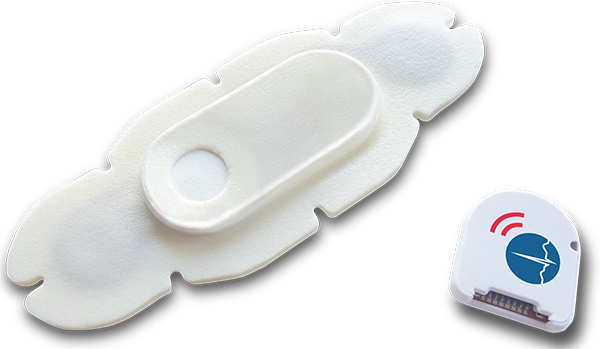


*Figure 1 HealthPatch sensor*
