# Supplementary material for: Do Simulated Hospital Admissions Reflect Reality? A Qualitative Study of Volunteer Well-Being During a 24-Hr Simulated Hospitalization
Source: HERD. 2021 Jun 9;14(4):130–46. doi: 10.1177/19375867211020682 (PMC8597193; doi:10.1177/19375867211020682)
Supplement: Supplemental Material, sj-docx-3-her-10.1177_19375867211020682 - Do Simulated Hospital Admissions Reflect Reality? A Qualitative Study of Volunteer Well-Being During a 24-Hr Simulated Hospitalization [file sj-docx-3-her-10.1177_19375867211020682.docx]

**Appendix 4: guideline semi-structured value-oriented interview**

General

1. Can you describe your experiences of the hospital admission of the past 24 hours?
2. Can you describe a few positive and a few negative experiences related to your admission?
3. What did you notice during your admission?
4. What were your expectations before the start of the admission?

Stress

1. How did you feel just before you entered the hospital?
2. Did you experience moments of stress or anxiety during your admission?
   1. If yes: when did you experience moments of stress?
   2. If yes: how did you deal with those stressful experiences? Did you try to distract yourself from the stressful experiences?

Sleep

1. How did you sleep during your admission?
2. How did you deal with this?
3. Do you have any recommendations on improving your quality of sleep during admission?

Activities

1. What did you do during the admission?
2. Did you feel restricted in your mobility during your admission?
   1. If yes: why did you feel restricted?
3. Do you have any recommendations on improving mobility during admission?
4. Did you feel bored during the admission?
   1. If yes: what would you have wanted to do? Do you have any recommendations for improvement?

Spatial comfort

1. What was your first experience when entering this patient room?
   1. What was your first experience when entering the bathroom?
2. What did you think about the view of the room?
3. Did you have all the necessary items in your room, or did you feel some items were missing?
4. What did you think of the atmosphere of the patient room?
   1. The sounds, the light, the temperature, the smell, the door open or closed,…?
5. What recommendations do you have on improving the patient room?

Privacy

1. How did you experience your privacy during admission?
2. What could improve your feelings of privacy?

Autonomy

1. How did you experience your autonomy during admission?
2. What could improve your feelings of autonomy?

Information provision

1. You were informed about the admission before this period started. Was this information clear?
   1. If not: what could have been improved?

Safety and Security

1. How did you experience your safety and security during admission?
2. What could improve your feelings of safety and security?

Social comfort

1. Did you receive a visit during your admission?
2. Did you try to contact other people at the ward (other patients, hospital personnel)?
   1. Why did / didn’t you do this? And how did you experience this?
3. How did you experience the contacts with the hospital personnel?
4. Do you have any recommendations on improving contact with hospital personnel?

‘Patient’ experience

1. Did you feel like a patient over the past 24 hours?
   1. Why did / didn’t you?
2. Do you have any recommendations for improving a simulated hospital admission?

Conclusion

1. Did we miss any topics you still would like to discuss?
2. Do you have any other remarks?

Thank you for your participation.
