# Supplementary material for: Do Simulated Hospital Admissions Reflect Reality? A Qualitative Study of Volunteer Well-Being During a 24-Hr Simulated Hospitalization
Source: HERD. 2021 Jun 9;14(4):130–46. doi: 10.1177/19375867211020682 (PMC8597193; doi:10.1177/19375867211020682)
Supplement: Supplemental Material, sj-pdf-1-her-10.1177_19375867211020682 - Do Simulated Hospital Admissions Reflect Reality? A Qualitative Study of Volunteer Well-Being During a 24-Hr Simulated Hospitalization [file sj-pdf-1-her-10.1177_19375867211020682.pdf]

My hospital experience

Room with a view

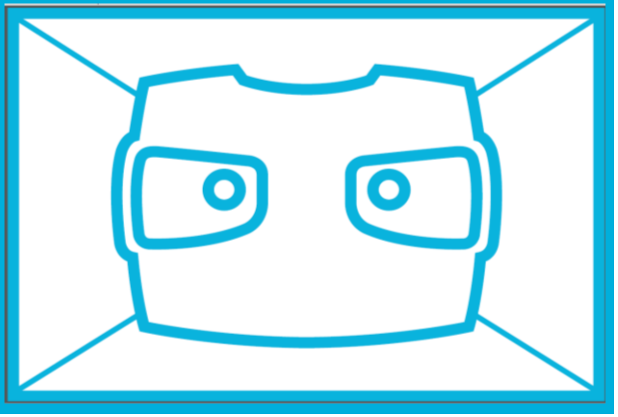

Radboudumc

Version 3, 10-07-2017

Introduction

Thank you for participating in this study!

This study is part of the research project “Room with a View”.
Room with a View aims at designing the ideal patient room for optimal patient well-being and experience. To do so, we aim to understand how patients and healthy volunteers experience an admission in the hospital.

We would like to ask you to reply to the questions in this booklet.
All answers are right, you cannot provide wrong answers. Try to describe your own experiences. You do not need to reply to questions for which you cannot provide an answer.

In case of questions, you can always contact us.

Personal details

This booklet belongs to:...

Draw or describe here who you are as a person.

Diary

What did I do today?

Today I did: ....

....

....

....

....

....

....

....

....

....

....

Who did I see?

Diary

Today I did: ....

What did I experience?

Diary

Tonight I did: ....

....

....

....

....

....

....

....

....

....

....

How did I sleep?

Diary

This morning I did: ....

....

....

....

....

....

....

....

....

....

....

Where did I go?

Listen...

What sounds do you hear? Walk around and describe the sounds you hear. Describe how you feel about these sounds.

1.I hear: ....
Location:..
I feel: *scared / angry / sad / confused / lonely / guilty / happy / content / neutral / different, namely:* ....
....

2.I hear: ....
Location: ....
I feel: ....

3.I hear: ....
Location: ....
I feel: ....

What do I think?

Complement or draw a circle around the words that matter for you during your hospitalization.

Privacy
Distraction
Friendliness
Music
Environment
……………………….
Clarity
Efficient
Sounds
Fast recovery
Sleep
Nature
Comfort
Being able to mobilize
……………………….
Own belongings
Art
Space for own responsibility
Contact with personnel
Day rooms
Light
Visitors
Contact with other patients
……………………….
A place to distract myself

Current hospital room

Draw or describe your current hospital room. Illustrate what you like and what you dislike about the room.

What do I think?

Ideal hospital room

Draw or describe your ideal hospital room.

Everything is allowed!

Fluids

Describe here what you have been drinking during your admission.

Time	What did you drink?	How much (ml)?

Fluids

Time	What did you drink?	How much (ml)?

Notes

Here is some space for additional notes and remarks.

Notes

Here is some space for additional notes and remarks.

Thank you for sharing!

A Room with a view

dreamproject
